# Immunomodulatory functions of ginsenosides in skin diseases: molecular mechanisms and therapeutic prospects

**DOI:** 10.3389/fphar.2026.1783370

**Published:** 2026-04-22

**Authors:** Donglin Yuan, Jiaxin Li, Zian Wang, Yu Han, Xue Yu, Zhendong Wei, Binbin Hou

**Affiliations:** 1 Department of Dermatology, The Second Hospital of Dalian Medical University, Dalian, Liaoning, China; 2 Department of Gastrointestinal Surgery, The Second Hospital of Dalian Medical University, Dalian, Liaoning, China

**Keywords:** atopic dermatitis, ginsenosides, melanoma, psoriasis, skin diseases

## Abstract

Ginsenosides, the primary bioactive constituents of *Panax ginseng* C.A.Mey. (Araliaceae), have attracted growing attention in dermatological research. A wide range of *in vitro* and *in vivo* studies has demonstrated that ginsenosides exert diverse biological effects encompassing anti-inflammatory, antioxidant, immunomodulatory, wound-healing, and antitumor activities. Given the multifactorial pathogenesis of most skin disorders, the multitarget potential of ginsenosides renders them particularly promising for cutaneous diseases. In this review, we summarize the chemical composition and classification of ginsenosides, as well as recent advances in their application against major skin conditions, including psoriasis, dermatitis, photoaging, chronic non-healing wounds, and skin melanoma. Mechanistically, ginsenosides modulate multiple signaling pathways related to inflammation, oxidative stress, skin barrier function, and tissue repair, such as NF-κB/NLRP3, PI3K/Akt/mTOR/MAPK, Nrf2/HO-1, and TGF-β/Smads. We also discuss the synergistic effects among different ginsenoside metabolites and their combined targeted interventions. Despite promising preclinical findings, challenges remain in clinical translation, such as low oral bioavailability and insufficient clinical evidence. Future studies should focus on novel delivery systems, precise target identification, and high-quality clinical trials to promote the development and application of ginsenoside-based therapies in dermatology.

## 1 Introduction

Panax ginseng C.A.Mey. [Araliaceae] is referred to as the “King of botanical drugs” in traditional medicine. It contains several pharmacologically active metabolites such as ginsenosides, phytosterols, polysaccharides and flavonoids (Fan et al., 2024; Park et al., 2018; Zhan et al., 2025). Ginsenosides, the primary active metabolites (Jia et al., 2025), are common and rare types (Wang et al., 2020), and both have antioxidant (Li et al., 2025), immunomodulatory and anti-inflammatory properties (Kim et al., 2017; Song et al., 2022; Wang et al., 2022). In recent decades, increasing experimental and clinical evidence has demonstrated that ginsenosides have neuroprotective and cardioprotective properties (Luo et al., 2019; Mohanan et al., 2018; Song et al., 2024; Tian et al., 2025; Zhang et al., 2021).

Skin is the largest organ of the body. It is constantly exposed to ultraviolet radiation, pollutants, pathogens and mechanical stress (Chambers and Vukmanovic‐Stejic, 2020; Zhang et al., 2022). Extensive exposure to these influences can accelerate skin aging and contribute to various skin diseases, including skin inflammation and degenerative skin diseases (Yang et al., 2025). Importantly, most dermatological diseases are multifactorial in nature, involving complex interactions among oxidative stress, immune dysregulation, barrier dysfunction, inflammatory signaling cascades, and aberrant cell proliferation. Therefore, therapeutic agents capable of modulating multiple molecular targets simultaneously may offer superior therapeutic potential compared to single-target interventions. In recent years, studies have shown that ginsenosides have anti-inflammatory, antioxidant, antimicrobial and wound-healing properties (Wang Y. et al., 2025; Yang et al., 2023), which provide a strong biological and pharmacological basis for their use in dermatological therapy (Liu E. et al., 2022; Lorz et al., 2020; Tian et al., 2023). Their ability to regulate diverse signaling pathways, including Nuclear factor kappa B (NF-κB), NOD-like receptor family pyrin domain-containing 3 (NLRP3) inflammasome, phosphoinositide 3-kinase (PI3K)/protein kinase B (Akt)/mechanistic target of rapamycin (mTOR), mitogen-activated protein kinase (MAPK), nuclear factor erythroid 2-related factor 2 (Nrf2)/heme oxygenase-1 (HO-1), and transforming growth factor-β (TGF-β)/Sma-and Mad-related proteins (Smads)-positions ginsenosides as promising multi-target modulators capable of addressing the complex pathophysiology of skin diseases (Cong et al., 2023; Kim et al., 2024). Nevertheless, despite increasing research interest, systematic and comprehensive investigations into the dermatological applications of ginsenosides remain scarce (Cong, et al., 2023). This integrative review summarizes the chemical structures, biological activities of ginsenosides, and their therapeutic potential in common skin disorders, including psoriasis, atopic dermatitis, photoaging, chronic non-healing wounds, fibrosis and melanoma, with a focus on their underlying molecular and cellular mechanisms.

In addition, we discuss some limitations and challenges that limit the translation of ginsenosides into clinical trials, such as limited bioavailability, variability in pharmacokinetic behavior, the lack of robust, well-designed clinical trials, and emerging strategies to overcome these challenges, such as drug delivery systems, structural optimization, and target identification. Finally, our goal is to provide a general conceptual framework that not only improves the understanding of the effects of ginsenosides on the skin, but also informs future prevention strategies, guides clinical decision-making, and stimulates the new development of ginsenoside-based skin therapies.

## 2 Chemical structure and classification of ginsenosides

Ginsenosides are triterpenoid saponins primarily obtained from the Panax species of the Araliaceae family. Based on structure of their aglycone backbones (Liu L. et al., 2020), ginsenosides are classified into three main classes: dammarane-type, oleanane-type (OA), and ocotillol-type (OCT) saponins (Geng et al., 2024; Hou et al., 2021), Among them dammarane saponins are the most abundant and studied class. Dammarane-type ginsenosides are further classified into protopanaxadiol-type (PPD) and protopanaxatriol-type (PPT) metabolites based on their differences in hydroxylation and glycosylation (Yu et al., 2023). PPD-type ginsenosides (including Rb1, Rb2, Rb3, Rc, Rd, F2, Rg3, Compound K (CK) and Rh2) are glycosylated at the C-3 and C-20 positions (Zhang et al., 2023). PPT-type ginsenosides, such as F1, Rg1, Rg2, and Re, have sugar moieties attached predominantly at the C-6 and C-20 positions (Cong, et al., 2023). These structural differences have significant effects on their physicochemical properties, metabolic fate and biological activity. OCT saponins have a unique epoxy structure with stereochemical and conformational properties (Nguyen et al., 2020), while OA saponins are based on oleanolic acid, indicating structural differences (Grabowska et al., 2024). Additionally, there are several malonylated saponins with glycosyl chains linked to malonyl groups (Hou, et al., 2021). This structural diversity supports the wide range of biological activities exhibited by ginsenosides (Minh Nguyen et al., 2020; Qu et al., 2021; Tang et al., 2021).

Although structural classification of ginsenosides into PPD and PPT subclasses is well established, comparative analysis of subclass-dependent pharmacological trends remains underemphasized. Emerging evidence suggests that structural differences influence membrane permeability, metabolic stability, and intracellular target accessibility (Choi et al., 2022; Wang Y. et al., 2024). In general, PPD-type metabolites, characterized by glycosylation at C-3 and C-20 positions, tend to exhibit greater cytotoxic and anti-proliferative effects in tumor models, possibly due to higher lipophilicity and improved cellular uptake. These features may partially explain their frequent evaluation in melanoma and other neoplastic systems (Han, Han, et al., 2020; Yang et al., 2016). By contrast, PPT-type metabolites, with glycosylation at C-6 and C-20 positions, are often associated with antioxidant and anti-inflammatory activities, particularly in models of photoaging and inflammatory dermatoses (Wang et al., 2026). The differing hydroxylation patterns may influence redox modulation and signaling pathway selectivity. However, systematic head-to-head comparisons under standardized conditions remain limited, and subclass-specific clinical positioning cannot yet be definitively established. Further subclass-guided pharmacological profiling is warranted.

It is important to note that many naturally occurring ginsenosides, particularly PPD-type saponins such as Rb1, Rc, and Rd, exhibit limited oral bioavailability due to their high molecular weight and glycosylation patterns (Li Y. et al., 2024; Yu et al., 2012). After oral administration, these macromolecular saponins are extensively metabolized by intestinal microbiota through stepwise deglycosylation to yield smaller and more absorbable metabolites (Chen et al., 2018), most notably CK (Shibata, 2001). In this context, several native ginsenosides may function pharmacologically as “prodrugs”, with CK representing a key bioactive metabolite responsible for systemic effects. This metabolic transformation introduces interindividual variability and has important implications for clinical translation.

## 3 Pharmacological effects of ginsenosides

### 3.1 Multi-pathway anti-inflammatory mechanisms of ginsenosides

Ginsenosides, the main bioactive metabolites of Panax species, have anti-inflammatory properties by modulating key signaling pathways involved in immune responses and inflammation (Guo C. et al., 2023; Jiang et al., 2025; Yi, 2021). Ginsenosides such as Rb1 and Rg3 suppress the NF-κB signaling pathway, leading to reduced expression of pro-inflammatory mediators, including Interleukin (IL)-4, Tumor necrosis factor alpha (TNF-α), Cyclooxygenase-2 (COX-2), and IL -1β (Han and Kim, 2020; Yu, et al., 2023). Rg1 was reported to target the NLRP3 inflammasome, an intracellular multiprotein complex responsible for the activation and release of pro-inflammatory proteins (Gao et al., 2020). Rg3 was further shown to regulate upstream transcriptional and hypoxia-related pathways, including mouse double minute 2 homolog (MDM2) and the transcription factor Hypoxia-inducible factor-1 alpha (HIF-1α), and thus reduces expression of thymic stromal lymphopoietin (TSLP), which plays a key role in initiating and amplifying allergic and immune-mediated inflammation (Han et al., 2021). Collectively, these results show that ginsenosides regulate anti-inflammatory responses simultaneously through transcription control, inflammation signaling and cytokine networks. This mechanistic breadth supports their potential for therapeutic use in systemic and dermatologic inflammatory disorders.

### 3.2 Antioxidant mechanisms of ginsenosides

Ginsenosides, the main bioactive metabolites of Panax species, are antioxidants that modulate multiple cell mechanisms involved in oxidative stress (He et al., 2022). Among these metabolites, Re activates the Nrf2 pathway, the central regulator of cell antioxidant defense. Upon activation, Nrf2 translocates to the nucleus and binds antioxidant response elements (AREs), promoting the transcription of enzymes such as HO-1 and NAD(P)H quinone oxidoreductase-1 (NQO-1). In addition, Rg1 exhibits free radical-scavenging capacity and reduces intracellular reactive oxygen species (ROS) (Moratilla-Rivera et al., 2023). Consequently, Rg1 protects dermal fibroblasts and epidermal keratinocytes from oxidative damage that contributes to photoaging, inflammation of the skin, and other ROS diseases (Piao et al., 2019). The stereoisomer Rg3 (S) has been shown to strengthen antioxidant defenses and reduce cell aging in human dermal fibroblasts by increasing mitochondrial peroxiredoxin-3 (PRDX3), while downregulating senescence-associated proteins including p16INK4a, p21Cip1 and p53, thus reversing cell aging and favoring a youthful cell state (Jang et al., 2020). Together, these studies demonstrate that ginsenosides preserve redox balance not only by directly neutralizing ROS but also by activating endogenous antioxidant pathways and modulating mitochondrial resilience. Such multi-layered regulation underlies their potential in mitigating oxidative stress-related skin aging and tissue damage.

### 3.3 Anti-tumor mechanisms of ginsenosides

Ginsenosides have been gaining attention for their anti-tumor properties across a wide range of cancers. Numerous studies show that different ginsenoside metabolites suppress tumor growth through cell cycle regulation, apoptosis, autophagy and immune modulation (Wang L. et al., 2025; Yang et al., 2024). For example, Rb1 was reported to induce S-phase arrest in lung cancer cell lines (95D and NCI-H460), thereby inhibiting proliferation (Feng et al., 2024). Rb1 also promoted mitochondrial-dependent apoptosis through modulation of p53, Bcl-2 related protein-x (Bax), and B-cell lymphoma 2 (Bcl-2) expression (Feng, et al., 2024). In triple-negative breast cancer models, Rh2 induced apoptosis by suppressing the IL-6/ Janus kinase 2 (JAK2)/signal transducer and activator of transcription 3 (STAT3) signaling pathway (Ding et al., 2024). Similarly, Rg3 inhibits prostate cancer cell growth by triggering apoptosis and cell cycle arrest via ROS (Peng et al., 2019). In colorectal cancer, Rg1 triggered autophagic cell death by inhibiting the Akt/mTOR pathway (Liu R. et al., 2024), Rd exerted antitumor activity in non-small cell lung cancer by activating the p53/Bax pathway, leading to S-phase arrest, mitochondrial apoptosis and reduction of tumor migration and invasion (Wan et al., 2024). Moreover, Rg5 directly binds to the catalytic site of PI3K, blocking its phosphorylation and downstream activation of Akt and mTOR pathways, leading to mitochondrial apoptosis and autophagy-associated cell death in MCF-7 breast cancer cells (Liu and Fan, 2020). In addition to direct cytotoxic effects, ginsenosides also enhanced anti-tumor immunity. In a murine MC38 colon cancer xenograft model, administered intragastrically with PD-L1 antibody activated the tyrosine kinase non-receptor 1 (TNK1)-interferon regulatory factor 3 (IRF3) signaling pathway, leading to increased tumor-derived C-X-C motif chemokine ligand 10 (CXCL10) production, increased CD8^+^ T cell invasion and improved PD-L1-targeted immunotherapy effectiveness (Huang et al., 2023).

Collectively, these findings indicate that ginsenosides suppress tumor growth through coordinated regulation of oncogenic and immune signaling networks, highlighting their potential as adjunctive candidates for skin cancers such as melanoma.

### 3.4 Immunomodulatory mechanisms of ginsenosides

Immunological studies have demonstrated that ginsenosides regulated both innate and adaptive immune responses across *in vitro* and *in vivo* models (You et al., 2022). In innate immunity, Rg1 enhanced macrophage activation by modulating PI3K/Akt/mTOR and NF-κB signaling, increasing TNF-α while reducing IL-6 (Wang et al., 2014). Rg3 promoted macrophage phagocytosis and TNF-α/IL-6 production via MAPK/AP-1 and NF-κB pathways, with ERK/c-Jun identified as a key mediator (Shin M.-S. et al., 2018; Xin et al., 2019). CK upregulated CD80 and CD86 in monocytes and macrophages through NF-κB and AP-1 activation, thereby enhancing phagocytic capacity (Kim and Cho, 2013; Yang et al., 2017). In adaptive immunity, Rg1 stimulated CD4^+^ T-cell proliferation and antigen-specific IgG production, whereas Rh2 increased CD4^+^/CD8^+^ T-cell infiltration and cytotoxicity in melanoma-bearing mice (Wang et al., 2017).

Recent results demonstrated that ginsenosides can effectively regulate the balance between T helper 1 (Th1) and T helper 2 (Th2) (Li et al., 2021). In a human ovalbumin sensitive BALB/c mouse model, ginsenoside Rg3 activated the Nrf2/HO-1 antioxidant signaling pathway and suppressed intercellular adhesion molecule-1 (ICAM-1) expression. This limited aberrant epithelial activation and leukocyte adhesion and infiltration. Thus, the local cytokine milieu shifted toward reduced Th2-associated cytokines (IL-4, IL-5, IL-13) and restored Th1-related interferon gamma (IFN-γ). Re-establishment of Th1/Th2 immune polarization attenuated eosinophilic inflammation, airway hyperresponsiveness, and mucus hypersecretion (Huang et al., 2021). Collectively, these immunomodulatory actions provide a mechanistic foundation for the therapeutic exploration of ginsenosides in immune-mediated dermatological conditions.

### 3.5 Neuroprotective mechanisms of ginsenosides

Ginsenosides exhibited neuroprotective properties by regulating oxidative stress, neuroinflammation, and neuronal survival pathways (Kong et al., 2024; Li Z.-Y. et al., 2024; Liu S. et al., 2024). Rg1 promoted axonal regeneration and functional recovery following nerve injury through upregulation of nerve growth factor (NGF) (Huo et al., 2015) and activated the JAK2/STAT3-vascular endothelial growth factor (VEGF) signaling axis, thereby supporting neurovascular remodeling (Yin et al., 2024). Other metabolites such as Re and Rd activated Nrf2/HO-1-mediated antioxidant responses and suppressed inflammatory pathways including NF-κB, PI3K, and MAPK, which reduced microglial activation and neuronal apoptosis (Chen et al., 2022; Qiao et al., 2022). Although these findings were primarily derived from neural injury and neurodegeneration models, several implicated pathways-particularly oxidative stress modulation and inflammatory signaling-overlap with mechanisms involved in neurogenic inflammation and chronic pruritus in dermatological disorders. Given that the skin functions as a neuro-immune interface, these shared molecular targets suggested a potential regulatory role of ginsenosides in neuro–cutaneous crosstalk. However, direct dermatological validation remained limited.

**FIGURE 1:**
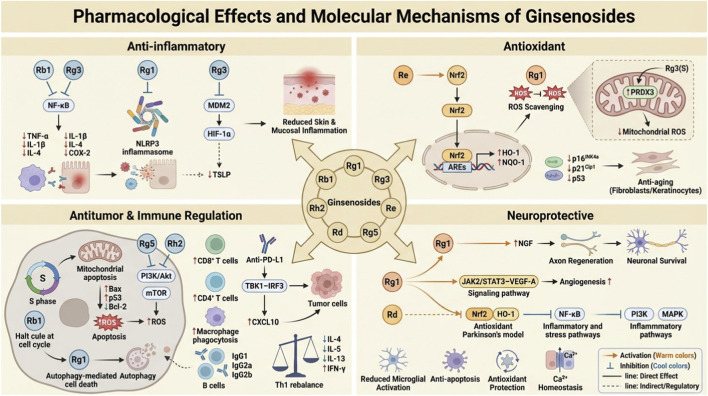
Pharmacological effects and molecular mechanisms of ginsenosides. This schematic overview summarizes the multi-target pharmacological activities and molecular mechanisms of major ginsenosides (Rb1, Rg1, Rg3, Rh2, Re, Rd, and Rg5). In the anti-inflammatory module, ginsenosides suppress inflammatory responses by inhibiting NF-κB signaling, NLRP3 inflammasome activation, and the MDM2/HIF-1α/TSLP axis, thereby reducing pro-inflammatory mediator production and alleviating skin and mucosal inflammation. In the antioxidant context, ginsenosides activate the Nrf2/ARE pathway, upregulate HO-1 and NQO-1, scavenge reactive oxygen species (ROS), and attenuate mitochondrial oxidative stress, contributing to protection against oxidative damage and skin aging. In antitumor and immune regulation, ginsenosides induce mitochondrial apoptosis, autophagy-mediated cell death, and cell cycle arrest via modulation of PI3K/Akt/mTOR and ROS-related pathways, while enhancing antitumor immunity through regulation of CD8^+^ T cells, macrophage phagocytosis, B cell responses, Th1/Th2 balance, and PD-1/PD-L1–associated signaling. In the neuroprotective module, ginsenosides promote angiogenesis and axon regeneration through the JAK2/STAT3–VEGF-A axis, enhance NGF-mediated neuronal survival, suppress inflammatory and stress-related pathways (including NF-κB and MAPK), reduce microglial activation, and maintain Ca^2+^ homeostasis.

Across the above sections, ginsenosides demonstrated coordinated multi-target regulatory effects, including anti-inflammatory, antioxidant, antitumor, immunomodulatory, and neuroprotective activities. Rather than acting on a single pathway, they converged on central signaling hubs such as NF-κB, Nrf2, PI3K/Akt/mTOR, JAK/STAT, and inflammasome networks, thereby regulating cytokine production, redox balance, immune polarization, epithelial integrity, and cell survival. These integrated mechanisms are schematically illustrated in Figure 1. Notably, many of these pathways are directly involved in the pathogenesis of inflammatory, immune-mediated, and neoplastic skin disorders, providing a mechanistic rationale for the dermatological applications discussed below.

## 4 Application of ginsenosides in the treatment of skin diseases

### 4.1 Ginsenosides as emerging therapeutics for psoriasis

Psoriasis is a chronic, relapsing inflammation of the skin due to immune dysregulation and characterized by erythematous, scaly papules and plaques (Guo J. et al., 2023). Conventional treatment options, such as topical corticosteroids, vitamin D3 antagonists, and systemic agents like acitretin, methotrexate, and cyclosporine, can provide benefits but often have side effects and a poor long-term tolerance (Ferrara et al., 2024; Lee and Kim, 2023). Although biologic treatment options have improved treatment outcomes and safety profiles (Spencer et al., 2023), their use in routine practice is restricted by limited response observed in some patients, injection site reactions, high cost and the risk of losing effectiveness over time (Rendon and Schäkel, 2019).

Recently, more attention has been drawn to the use of ginsenosides as alternative or adjunctive treatment of psoriasis. In imiquimod (IMQ)-induced murine models of psoriasis, oral administration of Rg1 reduced psoriasis-like skin inflammation by suppressing ROS/NLRP3 signaling. In particular, Rg1 inhibited the assembly and activation of the NLRP3 inflammasome, blocked cysteine-aspartic protease (caspase) −1 activation and decreased the production downstream of pro-inflammatory cytokines (IL-1β and IL-18) in keratinocytes (Mao et al., 2022). In the same model, Rg1 also suppressed NF-κB signaling. This effect was evidenced by reduced phosphorylation of IκBα and NF-κB p65, along with decreased secretion of IL-23, IL-17A, IL-1β, and TNF-α. In addition, Rg1 enhanced endogenous antioxidant defenses by increasing superoxide dismutase (SOD) activity and catalase activity and decreased malondialdehyde levels, a marker of lipid peroxidation. By modulating both the inflammatory and the oxidative pathways, Rg1 normalizes abnormal epidermal proliferation and improves Psoriasis Area and Severity Index (PASI) scores (Shi et al., 2019).

*In vitro* results demonstrated that CK suppresses the expression of regenerating islet-derived protein 3 alpha/gamma (REG3A/RegIIIγ) in keratinocytes and thus interrupts IL-36γ-driven inflammation. By this mechanism, CK reduces epidermal hyperplasia and excessive keratinization and improves IMQ-induced psoriasis skin lesions in murine models (Fan et al., 2019). Further *in vivo* studies showed that oral CK induced nuclear translocation of the glucocorticoid receptor (GR) and inhibited activation of NF-κB pathways. Therefore, the expression of key pro-inflammatory proteins in keratinocytes, including IL-6, TNF-α, CXCL-8, and ICAM-1, is markedly reduced, leading to alleviation of epidermal hyperplasia and local inflammation, and resulting in a significant improvement of psoriasis-like dermatitis in IMQ-treated mice (Wang W. et al., 2024). In addition to these results, further *in vitro* experiments showed that CK counteracts TNF-α/IFN-γ/VEGF165-induced keratinocyte hyperproliferation and disordered differentiation by activating GR and suppressing NF-κB signaling, which leads to downregulation of hyperproliferation-associated keratins K6, K16 and K17 and restoration of differentiation markers K1, K10 and loricrin. Furthermore, CK reduced the release of psoriasis-related inflammatory factors such as IL-6, TNF-α, CXCL-8 and ICAM-1, thereby contributing to normalization of keratinocyte function under inflammatory conditions (Wang W. et al., 2024).

In humanized psoriasis models, the AGR129 mice, a Rag2/Il2rg double knockout strain with severe combined immunodeficiency and prepsoriatic skin (PN skin), are used as skin graft recipients. Using Rh2 subcutaneously suppressed VEGF-A-mediated angiogenesis and led to a decrease in CD31-positive vascular density, decreased T-cell penetration and a thinner epidermis (Zhou et al., 2015). In addition, ginsenosides extracted from ginseng fruits (GFRS), including rare metabolites such as Rg3, ginsenoside Rk1, ginsenoside Rg5, ginsenoside F4 and ginsenoside Rg6, suppressed IL-17 signaling, suppressed IL-6/JAK/STAT pathway and downregulated NF-κB responses. These actions greatly reduced lipopolysaccharide(LPS)-induced inflammation in macrophages and keratinocytes, and decreased the production of nitric oxide (NO), IL-6, TNF-α and IL-17A. In mouse models of psoriasis, topical application of GFRS decreased IL-17A levels and alleviated erythema, scaling and epidermal thickening and improved the Psoriasis Area and Severity Index (PASI) score (Zheng et al., 2024). In summary, ginsenosides show promise as novel therapeutic agents for psoriasis due to their ability to suppress inflammation, inhibit the hyperproliferation of keratinocytes, and modulate immune responses.

### 4.2 Mechanistic insights of ginsenosides in atopic dermatitis treatment

Atopic dermatitis (AD) is a chronic, relapsing inflammatory skin disorder characterized by genetic predisposition, impaired epidermal barrier function, immune dysregulation, microbial imbalance, and environmental triggers (Sroka-Tomaszewska and Trzeciak, 2021). Conventional treatment relies on topical or systemic corticosteroids and immunosuppressants (which may provide relief from the symptoms, but are often limited by low efficacy and adverse effects associated with long-term use). Recently developed biologics and small molecules have improved the results and safety, but some patients still lack an adequate response and need new treatment to control the disease and patient quality of life.

Recent studies have shown that oral administration of F2 can ameliorate AD in murine models by altering gut microbiota composition and reducing serum immunoglobulin E (IgE) levels. In addition, F2 suppressed Th2-associated cytokines such as IL-4 and IL-13, as well as TNF-α (Li D. et al., 2024). This dual regulation of systemic IgE production and local inflammatory cytokines suggested a potential gut-skin axis-mediated immunomodulatory mechanism. Red ginseng extract (RGE) enriched in ginsenosides such as Rb1, Rg1, Rg3, and Rh1(Samukawa et al., 2012) has been shown to be therapeutically beneficial in 1-fluoro-2,4 dinitrobenzene (DNFB)-induced AD mouse model. Topical application of RGE inhibited phosphorylation of p70S6K in skin lesions, reducing inflammation like ear swelling and pruritus (Osada-Oka et al., 2018). *In vitro* experiments demonstrated that RGE and its metabolites Rg3 and Rh1 inhibited p70S6K phosphorylation in IgE-stimulated human basophils (KU812) and IFN-γ-stimulated human keratinocytes (NHEK). This inhibition downregulated chemokine ligand 2 (CCL2) expression and reduced the release of pro-inflammatory cytokines, thereby attenuating inflammatory and allergic responses associated with chronic AD (Osada-Oka, et al., 2018). These findings indicate that ginsenosides may modulate immune cell-keratinocyte interactions central to chronic AD inflammation.

Barrier dysfunction is a defining feature of AD pathogenesis, CK is applied on a 2,4-dinitrochlorobenzene (DNCB) induced AD mouse model to increase expression of serine protease inhibitor Kazal type 5 (SPINK5) and KLK5-PAR-2 pathway (KLK5)-activated receptor-2 (PAR2) signaling. By suppressing KLK5, CK prevented degradation of desmosomal proteins like desmocollin-1, reconstructed desmosomal structures, claudin-1 and ZO-1, thereby improving intercellular adhesion and tight junction integrity. CK treatment reduced transepidermal water loss and improved skin barrier function (Park et al., 2020). In AD model induced by MC903 (calcipotriol), Rg1, matrine powder and icariin together inhibited IgE type I hypersensitivity. CD4^+^ T cells in the lesion area, tight junction proteins ZO-1 and claudin-1, skin inflammation and barrier dysfunction are also improved by combination treatment. The effect of this metabolite was comparable to that of dexamethasone but not to the growth inhibition or hepatorenal toxicity (Wu et al., 2025). However, the specific contribution of Rg1 within combination regimens requires further clarification.

In the NC/Nga mouse model with spontaneous AD-like lesions, Rh2 reduced TSLP expression in keratinocytes through NF-κB inhibition. This effect suppressed Th2-cell differentiation and effector function, decreased GATA binding protein 3 (GATA3) expression, and reduced the number of IL-4–producing Th2 cells. Thus, epidermal thickening, mast cell and eosinophil infiltration and serum IgE levels were significantly reduced. Rh2 has better therapeutic effect than dexamethasone without a growth inhibition or immune organ expansion (Ko et al., 2019).

Patients with AD often have IgE-mediated allergic reactions (Hill and Spergel, 2018). Ginseng leaf extract (GLE) contains seven ginsenosides, including Rb3, Rd, Re, Rb2, Rc, Rb1, and Rg1. In DNCB-induced murine AD, intraperitoneal administration of GLE suppressed NF-κB and NLRP3 activation and MAPK signaling. The treatment suppressed mast cell degranulation and reduces the release of histamine, IgE, ROS and several pro-inflammatory cytokines. GLE alleviated mast cell–mediated allergic inflammation, improved skin lesions and delayed the development and progression of atopic comorbidities. No toxic effects were observed during the treatment period (Oh et al., 2024).

Collectively, these findings suggest that ginsenosides intervene at multiple core levels of AD pathogenesis rather than merely suppressing downstream inflammation. They modulate epithelial alarmins (e.g., TSLP), rebalance Th1/Th2 polarization, attenuate IgE-driven responses, and restore barrier integrity. This multi-compartment regulation across immune, epithelial, and structural axes distinguishes ginsenosides from single-target anti-inflammatory agents. Such integrated modulation provides a mechanistic rationale for their potential role as adjunctive strategies in AD management, pending clinical validation.

### 4.3 Mechanistic insights into the anti-photoaging effects of ginsenosides

Skin photoaging, chronic dermal damage caused by prolonged ultraviolet (UV) exposure, leads to wrinkles, hyperpigmentation, and loss of skin elasticity (Marshall et al., 2025). UV radiation disrupts dermal extracellular matrix homeostasis by causing oxidative stress, inflammatory process, degradation of collagen, and accelerates skin age (Liu X. Y. et al., 2022; Wan et al., 2021). Recent studies have shown that different ginsenosides intervened at different stages of this process, promising approaches for photoaging prevention and treatment.

In ultraviolet radiation B (UVB)-induced photoaging experiments using human immortalized keratinocytes (HaCaT), Rk1 reduced ROS accumulation and malondialdehyde (MDA) peroxidation products, decreased inflammation (IL-6), downregulated matrix metalloproteinases (MMPs), including MMP-3 and MMP-9 expression, and increased type I and III collagen (Liu Y. et al., 2022). In UVB-exposed BALB/c nude mice, daily topical application of Rk1 to the dorsal skin decreased activation of the PI3K/Akt/NF-κB signaling pathway. This treatment reduced epidermal thickening, prevented collagen fiber disorganization, and attenuated wrinkle formation (Liu Y. et al., 2022).

Similarly, an Rg3-loaded thermosensitive gel (P407/CS/HA) reduced ROS and MDA production and restored antioxidant enzyme activities, including SOD and glutathione peroxidase (GSH-Px). The formulation suppressed the NF-κB/COX-2 and inducible nitric oxide synthase (iNOS) inflammatory pathways, downregulated pro-apoptotic proteins Bax and caspase-3, and upregulated the anti-apoptotic protein Bcl-2. These coordinated effects mitigated oxidative stress and inflammation. Together, these actions strengthen the skin’s antioxidant defense system and retard photoaging (Ding et al., 2023).

Reduction in collagen synthesis is a hallmark of skin photoaging. In UVB-exposed human dermal fibroblasts (NHDFs), ginsenoside C-Mc activated the TGF-β/Smad signaling pathway, enhanced Smad2/3 phosphorylation, and suppressed Smad7 expression, thereby restoring type I procollagen synthesis and improving dermal matrix integrity. Concurrently, modulation of the MAPK/AP-1/NF-κB axis reduced MMP-1 and MMP-3 expression, limiting extracellular matrix degradation. Through coordinated regulation of collagen production and matrix remodeling pathways, C-Mc mitigated UVB-induced photoaging-associated structural damage (Liu X. Y. et al., 2022). In the UVB-irradiated NHDF model, ginsenoside C-Y reduced ROS levels, MMP-1 expression, and the production of pro-inflammatory cytokines, including IL-6, TNF-α, and VEGF. C-Y also suppressed the MAPK/Activator Protein-1 pathway, thereby limiting collagen degradation, and enhancing type I procollagen production (Liu et al., 2019). In melanocyte models (Melan-a cells) and zebrafish embryos C-Y downregulated key melanogenesis regulators (microphthalmia-associated transcription factor (MITF), tyrosinase, tyrosinase-related protein 1 (TRP-1), tyrosinase-related protein 2 (TRP-2)), inhibiting melaninsynthesis (Liu, et al., 2019).

In ultraviolet A (UVA)-induced photoaging models using HaCaT keratinocytes, CK combined with retinol and its derivatives enhanced antioxidant defenses, inhibited apoptosis, promoted collagen production, prevented extracellular matrix degradation, and attenuated cellular senescence (Zhang et al., 2025).

Together, these results show that ginsenosides counteract key photoaging mechanisms by multi-target antioxidant, anti-inflammatory and collagen-regulating effects, which can be used to develop effective treatments against skin photoaging.

### 4.4 Mechanistic insights into the wound-Healing and anti-fibrotic actions of ginsenosides

Emerging evidence indicates that ginsenosides promote wound repair through coordinated regulation of keratinocyte migration, angiogenesis, extracellular matrix remodeling, and fibrosis-related signaling pathways.

Rb1 was shown to enhance keratinocyte migration in scratch assays by modulating sphingosine-1-phosphate (S1P) metabolism through the upregulation of sphingosine kinase 1 (SPHK1) and the inhibition of S1P lyase, leading the activation of extracellular signal-regulated kinase 1/2 (ERK1/2) and NF-κB signaling and increased MMP-2 and MMP-9 expression (Shin K. O. et al., 2018). Further work showed that Rb1 activated the p38MAPK/MSK2/NF-κB signaling pathways and increased senescence associated secretory phenotype (SASP) in senescent dermal cells. Rb1 selectively regulated repair factors such as platelet-derived growth factor (PDGF), TGF-β1, and VEGF and promoted the migration and differentiation of myofibroblasts (Hou and Kim, 2018).

In a clinical study of patients with keloids, Rg3 inhibited the TGF-β/Smad pathway and reduced angiogenic mediators, including VEGF and CD31. This suppression limited fibroblast proliferation, extracellular matrix deposition (type I and III collagen), and neovascularization while promoting apoptosis of keloid cells, suggesting anti-fibrotic potential (Tang et al., 2018).

In diabetic wound models, Rg1 accelerated healing by inhibiting miR-489-3p and upregulating sirtuin 1 (Sirt1), which subsequently activated PI3K/Akt and endothelial nitric oxide synthase (eNOS) signaling, improving endothelial function and angiogenesis (Qin et al., 2025). Rg1 also restored migration and adhesion of high-glucose–impaired human umbilical vein endothelial cells (HUVECs) and reduced oxidative stress–associated apoptosis, further supporting vascular repair and burn wound recovery (Qin, et al., 2025). Collectively, these findings demonstrate that ginsenosides regulate multiple interconnected pathways governing migration, angiogenesis, inflammation, and fibrosis, thereby supporting both tissue regeneration and scar modulation.

### 4.5 Mechanistic insights into the anti-melanoma effects of ginsenosides

Cutaneous melanoma is a common skin cancer with poor prognosis and limited treatment options. It can spread through lymph nodes and blood in the early stages and is poor in terms of prognosis and treatment results (Tímár and Ladányi, 2022). Although targeted therapies, chemotherapy, and immune checkpoint inhibitors have improved survival in some patients, treatment resistance and incomplete responses remain major challenges. In this context, ginsenosides derived from Panax ginseng have been investigated for their potential multi-target anti-melanoma effects, though the strength and translational relevance of the current evidence require careful evaluation (Kim et al., 2019). *In vitro* studies reported that Rg3 induced apoptosis in human A375.S2 cells by increasing the Bax/Bcl-2 ratio and attenuating the mitogen-activated protein kinase (MEK) -ERK pathway activity. In B16F10 cells, Rg3 suppressed ERK/Akt/mTOR signaling activity, reduced expression of proliferation-related proteins including proliferating cell nuclear antigen (PCNA), MMP-2/9, and VEGF, and induced S-phase cell cycle arrest (Meng et al., 2019). Rg3 also inhibited epidermal growth factor receptor (EGFR)/ERK pathway activity by downregulating fucosyltransferase 4 (FUT4), leading to significant inhibition of tumor cell proliferation in A375 cells (Shan et al., 2015).

Rh2 is a broad-spectrum anti-melanoma agent targeting the non-receptor tyrosine kinase proto-oncogene tyrosine-protein kinase Src (Src). Rh2 inhibited Src/STAT3 signaling activity in A375 and B16F10 cells, reducing STAT3 Tyr705 phosphorylation and nuclear translocation, which led to decreased expression of myeloid cell leukemia 1 (Mcl-1), Bcl-xL, and Cyclin D1, ultimately inducing G0/G1 arrest and mitochondrial apoptosis (Li J. K. et al., 2024). In a murine B16F10 melanoma model, intraperitoneal Rh2 administration increased intratumoral CD4^+^ and CD8a^+^ T cells and enhanced splenic lymphocyte cytotoxicity against B16F10 cells. This resulted in dose-dependent tumor growth inhibition and prolonged survival (Wang, et al., 2017). Rh2 combined with the PIM-1 inhibitor SMI-4a potentiated tumor suppression in an A375 xenograft model through inhibition of AKT/mTOR signaling activity and increased expression of LC3-II and Beclin-1. The combination therapy promoted autophagy-mediated cell death. *In vitro* experiments further demonstrated that Rh2 pretreatment combined with SMI-4a increased LC3-II expression in A375 and G361 cells, activated caspase-3/7, and reduced tumor cell viability (Lv et al., 2018).

Re suppressed melanoma cell proliferation through modulation of AKT/ERK signaling pathway. Independently, Re promoted ubiquitin-mediated degradation of MITF in B16F10 cells, contributing to apoptosis induction and tumor growth inhibition. In a subcutaneous B16F10 tumor model in C57BL/6 mice, Re administration downregulated MITF and its downstream targets Bcl-2 and HIF-1α, accompanied by increased the number of cleaved caspase-3–positive cells, induced tumor cell death, suppressed Ki67-associated proliferation, and promoted vascular normalization. *In vitro* studies further showed that Re competitively inhibited tyrosinase activity in B16F10 cells and reduced both intracellular and extracellular melanin levels. It also suppressed α-melanocyte-stimulating hormone (α-MSH)–induced melanogenesis by downregulating MITF, tyrosinase, and TRP-1/2 expression. These effects were accompanied by reduced melanoma cell proliferation, increased apoptosis, and decreased pigment production (Hwang et al., 2023).

Pharmacological studies have shown that ginsenoside Ro can be readily metabolized by gut microbiota *in vitro*, producing 3 main metabolites: IVazingibroside R1, Bambusosaponin IVa and Calenduloside E. Among these metabolites, Calenduloside E exhibited the strongest anti-melanoma activity, with a half maximal inhibitory concentration (IC50) of 2.58 μg/mL and 85.4% growth inhibition in B16F10 cells. *In vitro* angiogenesis assays further demonstrated that these metabolites inhibited lumen formation by HUVECs and disrupted neovascularization and vascular abnormalities in tumor models. This dual activity on tumor cells and vascular structures may contribute to the overall anti-melanoma effects attributed to Ro (Zheng et al., 2019). In addition, Rk1 activated both intrinsic and extrinsic apoptotic pathways in SK-MEL-2 human melanoma cells. Its cytotoxic effects were dose-dependent and appeared greater than those of Rg3, while exhibiting comparatively low toxicity toward normal cells (Kim et al., 2012).

Overall, ginsenosides modulated multiple oncogenic pathways to induce apoptosis, inhibit angiogenesis, and suppress melanoma cell proliferation. These multi-target effects, coupled with relatively low experimental toxicity, warrant further mechanistic and translational evaluation.

Collectively, across major dermatological disorders, ginsenosides modulated shared pathogenic hubs involving oxidative stress, inflammatory signaling, immune polarization, angiogenesis, and extracellular matrix remodeling. By converging on pathways such as NF-κB, NLRP3, PI3K/Akt/mTOR, MAPK, and TGF-β/Smad, these metabolites exerted multi-layer regulatory effects rather than single-target actions. However, given the molecular redundancy and complexity of chronic skin diseases, intervention by a single ginsenoside may be insufficient to fully disrupt disease-driving networks. This provides a mechanistic basis for exploring combinatorial strategies among ginsenoside metabolites.

**FIGURE 2:**
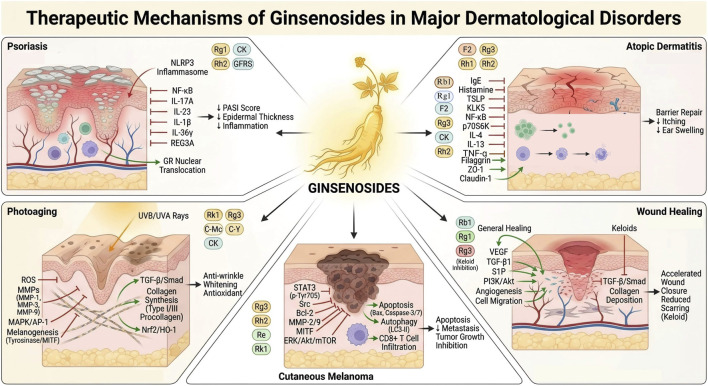
Therapeutic mechanisms of ginsenosides in major dermatological disorders. This schematic summarizes the therapeutic mechanisms of ginsenosides in psoriasis, atopic dermatitis, photoaging, wound healing and fibrosis, and cutaneous melanoma. In psoriasis, ginsenosides attenuate inflammation by inhibiting NLRP3 inflammasome activation and NF-κB signaling, reducing pro-inflammatory cytokines, promoting glucocorticoid receptor (GR) nuclear translocation, and improving epidermal hyperplasia and PASI scores. In atopic dermatitis, ginsenosides suppress IgE-mediated and Th2-dominant responses via inhibition of NF-κB, MAPK, TSLP and KLK5 related signaling, enhance epidermal barrier proteins. In photoaging, ginsenosides protect against UVA/UVB-induced skin damage by reducing oxidative stress, inhibiting MMP and MAPK/AP-1-mediated collagen degradation, activating Nrf2/HO-1 and TGF-β/Smad pathways, promoting collagen synthesis, and suppressing melanogenesis. In wound healing, ginsenosides promote cell migration and angiogenesis via S1P-, VEGF-, PI3K/Akt -dependent pathways, while inhibiting TGF-β/Smad-driven collagen deposition and scar formation. In cutaneous melanoma, ginsenosides inhibit tumor growth by suppressing Src/STAT3, ERK/Akt/mTOR and MITF signaling, inducing apoptosis and autophagy-mediated cell death, reducing angiogenesis, enhancing antitumor immunity, and inhibiting melanogenesis.

**FIGURE 3:**
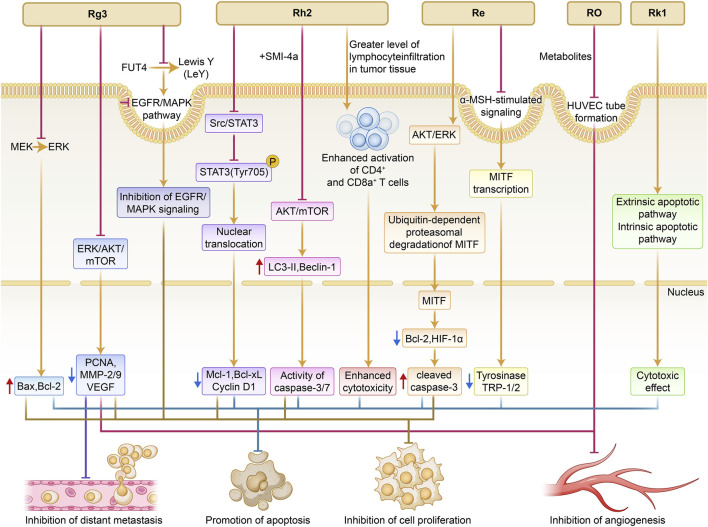
Schematic of multi-pathway anti-tumor mechanisms of ginsenosides in cutaneous melanoma. The figure illustrates key molecular pathways targeted by representative ginsenosides in melanoma. Rg3 inhibits MEK/ERK and EGFR/MAPK signaling, reducing metastatic markers. Rh2 suppresses Src/STAT3 signaling, enhances autophagy, and promotes CD4^+^/CD8^+^ T-cell–mediated cytotoxicity. Re induces ubiquitin-dependent degradation of MITF, thereby inhibiting melanogenesis and cell proliferation. Ro metabolites block endothelial tube formation, limiting angiogenesis. Rk1 activates intrinsic and extrinsic apoptotic pathways, enhancing melanoma cell death. Together, these ginsenosides exert multi-target inhibitory effects on melanoma growth, survival, and dissemination.

The therapeutic applications of ginsenosides across major dermatological disorders and their corresponding mechanistic axes are summarized in Figure 2. Multi-pathway anti-tumor mechanisms of ginsenosides in cutaneous melanoma are summarized in Figure 3. The representative molecular mechanisms of major ginsenosides across different dermatological disorders are summarized in Table 1. The detailed evidence appraisal is provided in Supplementary Table S1.

**TABLE 1:** Representative mechanisms of ginsenosides in various skin diseases.

Ginsenoside disease	Psoriasis	Atopic dermatitis	Skin photoage	Skin healing	Skin melanoma
Rg1	Reduce the release of Th1 and Th17-type inflammatory factors, reverse abnormal epidermal cell proliferation, aimprove PASI scores (Mao et al. 2022), (Shi et al. 2019)	Block Th2-type inflammatory factor expression, reduce serum IgE levels, alleviate skin lesions and pruritus (Wu et al. 2025))	​	Enhance vascular endothelial cell function and promote angiogenesis; restore the migration and adhesion capacity of HUVECs, inhibit oxidative stress and apoptosis, promote burn wound repair (Qin et al. 2025)	​
Rh2	Inhibit VEGF-A-mediated angiogenesis, reduce CD31-positive vascular density, decrease T-cell infiltration and epidermal thickness, improves PSO lesions (Zhou et al. 2015)	Modulate Th2 cell differentiation and function, inhibit mast cell and eosinophil infiltration, reduce serum IgE levels, alleviate AD epidermal hyperplasia (Ko et al. 2019)	​	​	Induce G0/G1 phase arrest and activate mitochondrial apoptosis, demonstrate broad-spectrum antineoplastic activity against melanoma (Li J. K., et al. 2024). Enhance CD4⁺ and CD8a⁺ T cell infiltration within tumors, boost splenic lymphocyte cytotoxicity, inhibit tumor growth (Wang, et al. 2017). Inhibit AKT/mTOR signaling and induce autophagy (↑LC3-II/Beclin-1), activate caspase-3/7, suppress melanoma cell viability and tumor growth (Lv et al. 2018)
CK	Block the excessive proliferation and abnormal differentiation of keratinocytes, inhibit the release of psoriasis-associated inflammatory factors, alleviate psoriasis-like skin lesions (Fan et al. 2019), (Wang Y., et al. 2024)	Restore the integrity of the desmosome structure, enhance tight junction protein levels, reduce transepidermal water loss (TEWL) to improve skin barrier function (Park et al. 2020)	Accelerate cell proliferation, upregulate collagen and elastin expression, inhibit MMPs activity, alleviate oxidative stress, thereby reversing cellular senescence phenotype (Zhang et al. 2025)	​	​
F2	​	Regulate gut microbiota, reduce IgE levels and Th2 pro-inflammatory factors, alleviate skin inflammation (Li D. et al., 2024)	​	​	​
GLE	​	Reduce the release of histamine, IgE, ROS, and multiple pro-inflammatory factors, inhibit mast cell-mediated allergic inflammation, alleviate skin lesions (Hill and Soergel, 2018)	​	​	​
GFRS	Inhibit inflammatory responses in macrophages and keratinocytes, reduce expression of Th1 and Th17-type psoriasis-associated inflammatory factors, improve skin erythema, scaling, and thickening (Zheng et al. 2024)	​	​	​	​
RGE	​	Blocke p70S6K phosphorylation, inhibit the mRNA expression of downstream chemokine CCL2, thereby reducing inflammatory mediator release and exerting anti-inflammatory and anti-allergic effects (Osada-Oka, et al. 2018)	​	​	​
Rk1	​	​	Reduce inflammatory factors such as IL-6, downregulates MMP-3/9 expression, elevate type I and III collagen levels, alleviate epidermal thickening, collagen fiber disorganization, and wrinkle formation (Liu Y. et al. 2022)	​	Activate exogenous and endogenous apoptosis pathways to promote cell apoptosis (Kim et al. 2012)
Rg3	​	​	Reduce oxidative stress, suppress inflammatory responses, prevent apoptosis, enhance the skin's antioxidant defense capacity, delay photoaging (Ding et al. 2023)	Inhibit the proliferation, migration, invasion, and neovascularization of fibroblasts (KFs), reduce the deposition of type I/III collagen and extracellular matrix (ECM), induce apoptosis, and block the expansion of keloids (Tang et al. 2018)	Induce apoptosis in melanoma A375.S2 cells; block S phase progression in B16-F10 cells, thereby inhibiting their proliferation, invasion, and metastasis; inhibit the proliferation of human malignant melanoma A375 cells (Meng et al. 2019), (Shan et al. 2015)
C-MC	​	​	Activate the TGF-β/Smad signaling pathway promote Smad2/3 phosphorylation, thereby reversing UVB-induced inhibition of type I collagen synthesis (Liu X. Y. et al., 2022).	​	​
C-Y	​	​	Inhibit the release of ROS, MMP-1, VEGF, and Th1-type inflammatory factors, reduce collagen degradation, promote type I procollagen synthesis, thereby alleviating photoaging damage (Liu et al. 2019)	​	​
Rb1	​	​	​	Selective upregulation of reparative factors, accelerate keratinocyte migration and induce myofibroblast differentiation, thereby promoting skin wound healing (Shin K. O. et al. 2018), (Hou and Kim, 2018)	​
Re	​	​	​	​	Modulate the expression of MITF, tyrosinase and TRP-1/2. Inhibit melanoma cell proliferation, induce apoptosis, block pigment synthesis (Hwang et al. 2023)
Ro	​	​	​	​	Block angiogenesis and vascular remodeling in the tumor microenvironment to exert anti-melanoma effects (Zheng et al. 2019)

## 5 Current research on the combination of ginsenoside metabolites

Recent studies suggest that single ginsenoside metabolites may not fully address the multifactorial nature of complex diseases. This has prompted increasing interest in potential synergistic or complementary effects among multiple ginsenoside metabolites. Evidence from cardiovascular, metabolic, immune, and inflammatory models demonstrated that specific ginsenoside pairs-such as Rb2/Rg3 (Choi et al., 2021), Rb3/Rb2 (Liu X. et al., 2020), Rb1/Rb2 (Go et al., 2020), (24R)-pseudo-ginsenoside HQ (R-PHQ)/(24S)-pseudo-ginsenoside HQ (S-PHQ) (Qi et al., 2019), and Rg2/Rh1 (Huynh et al., 2020)-modulated key signaling pathways, including PI3K/Akt, MAPK/ERK, Akt/mTOR, and Toll-like receptor 4 (TLR4)–NF-κB/STAT1. These coordinated interactions were associated with enhanced angiogenesis, reduced mitochondrial-dependent apoptosis, improved myogenic differentiation, restoration of immune balance, and decreased production of pro-inflammatory cytokines.

Although most existing evidence is not from a skin system, the mechanistic results highlight that ginsenoside combinations have multi-target network-level regulatory effects. These properties provide a theoretical basis for studying ginsenoside combinations in dermatology for chronic inflammation, oxidative damage, immune dysregulation, and abrasion.

Overall, these results show that ginsenoside combinations have a more complete and integrated regulatory profile than single metabolites, especially in inflammation, oxidative stress and tissue repair pathways. Further studies and human clinical trials for ginsenoside-based therapies are needed to validate their safety and efficacy.

## 6 Summary

Ginsenosides are the main pharmaceutically active metabolites of Panax ginseng and are often studied by dermatologists due to their varied biological activities. Experimental evidence suggests that these triterpenoid saponins have anti-inflammatory, antioxidant, immunomodulatory, wound healing and antitumor properties, which makes them promising candidates for many skin diseases. In this review, we summarize existing work, including chemical classification, pharmacokinetic and biological properties of ginsenosides, and evaluate their therapeutic potential for major skin diseases such as psoriasis, dermatitis, photoaging, chronic wounds, and melanoma.

The molecular level, ginsenosides control complex networks of signaling pathways. They inhibit NF-κB activation and NLRP3 inflammasome assembly, reprogram the PI3K/Akt/mTOR and MAPK signaling pathways, activate Nrf2/HO-1 antioxidant pathways and regulate TGF-β/Smad signaling pathways for extracellular matrix remodeling and tissue repair. These coordinated actions reduce inflammation, minimize oxidation, restore epidermal barrier function, and promote tissue regeneration. Recent studies suggest that using multiple ginsenoside metabolites together can improve therapeutic effectiveness by multitarget synergistic effect, while minimizing any negative effects, modernizing traditional Chinese medicine.

While early experimental findings are encouraging, it is important to note that the majority of current evidence derives from *in vitro* and animal models, and robust, large-scale randomized clinical trials remain scarce. The principal translational obstacle for ginsenosides remains limited and highly variable bioavailability. Many native ginsenosides are macromolecular saponins with poor intestinal permeability and extensive first-pass metabolism, and their systemic activity often depends on gut microbiota–mediated deglycosylation into more absorbable metabolites such as CK. This microbiota-dependent biotransformation introduces interindividual variability and complicates dose standardization.

Future research should therefore prioritize: (i) development of microbiota-informed prodrug strategies and direct CK-based formulations; (ii) nano-delivery systems, lipid-based carriers, and dermal-targeted formulations to enhance stability and tissue penetration; (iii) systematic pharmacokinetic profiling under standardized dosing paradigms; and (iv) stratified clinical trials integrating metabolic phenotyping to address response heterogeneity. Addressing these pharmacokinetic and translational constraints will be essential for moving ginsenoside-based therapeutics from mechanistic plausibility toward evidence-based dermatological application.

## Glossary

OA: oleanane-type
OCT: ocotillol-type
PPD: Protopanaxadiol
PPT: Protopanaxatriol
CK: Compound K
NF-κB: Nuclear factor kappa B
IL: Interleukin
TNF-α: Tumor necrosis factor alpha
COX-2: Cyclooxygenase-2
NLRP3: NOD-like receptor family pyrin domain-containing 3
HIF-1α: Hypoxia-inducible factor-1 alpha
TSLP: thymic stromal lymphopoietin
Nrf2: nuclear factor erythroid 2-related factor 2
HO-1: heme oxygenase-1
ROS: reactive oxygen species
Bax: Bcl-2 related protein-x
Bcl-2: B-cell lymphoma 2
JAK2: Janus kinase 2
STAT3: signal transducer and activator of transcription 3
PI3K: phosphoinositide 3-kinase
Akt: protein kinase B
mTOR: mechanistic target of rapamycin
MAPK: mitogen-activated protein kinase
Th1: T helper 1
Th2: T helper 2
ICAM-1: intercellular adhesion molecule-1
SOD: superoxide dismutase
GR: glucocorticoid receptor
IFN-γ: interferon gamma
AD: Atopic dermatitis
GFRS: ginsenosides extracted from ginseng fruits
RGE: Red ginseng extract
IgE: immunoglobulin E
DNFB: 1-fluoro-2,4 dinitrobenzene
DNCB: 2,4-dinitrochlorobenzene
GLE: ginseng leaf extract
UV: ultraviolet
MMPs: metalloproteinases
UVB: ultraviolet radiation B
GSH-Px: glutathione peroxidase
MITF: microphthalmia-associated transcription factor
S1P: sphingosine-1-phosphate
VEGF: vascular endothelial growth factor
Sirt1: sirtuin 1
HUVECs: human umbilical vein endothelial cells
Src: proto-oncogene tyrosine-protein kinase Src.

## References

[B1] Chambers E. S. Vukmanovic‐Stejic M. (2020). Skin barrier immunity and ageing. Immunology 160, 116–125. 10.1111/imm.13152 31709535 PMC7218662

[B2] Chen M.-Y. Shao L. Zhang W. Wang C.-Z. Zhou H.-H. Huang W.-H. (2018). Metabolic analysis of Panax notoginseng saponins with gut microbiota-mediated biotransformation by HPLC-DAD-Q-TOF-MS/MS. J. Pharmaceutical Biomedical Analysis 150, 199–207. 10.1016/j.jpba.2017.12.011 29245089

[B3] Chen Y. Y. Liu Q. P. An P. Jia M. Luan X. Tang J. Y. (2022). Ginsenoside Rd: a promising natural neuroprotective agent. Phytomedicine. Jan. 95, 153883. 10.1016/j.phymed.2021.153883 34952508

[B4] Choi R. J. Mohamad Zobir S. Z. Alexander-Dann B. Sharma N. Ma M. K. L. Lam B. Y. H. (2021). Combination of ginsenosides Rb2 and Rg3 promotes angiogenic phenotype of human endothelial cells via PI3K/Akt and MAPK/ERK pathways. Front. Pharmacol. 12, 618773. 10.3389/fphar.2021.618773 33643049 PMC7902932

[B5] Choi H. S. Koo H. B. Jeon S. W. Han J. Y. Kim J. S. Jun K. M. (2022). Modification of ginsenoside saponin composition via the CRISPR/Cas9-mediated knockout of protopanaxadiol 6-hydroxylase gene in Panax ginseng. J. Ginseng Res. 46, 505–514. 10.1016/j.jgr.2021.06.004 35818421 PMC9270645

[B6] Cong L. Ma J. Zhang Y. Zhou Y. Cong X. Hao M. (2023). Effect of anti-skin disorders of ginsenosides-A systematic Review. J. Ginseng Res. 47, 605–614. 10.1016/j.jgr.2023.04.005 37720567 PMC10499590

[B7] Ding C. Peng X. Yang J. Chen K. Liu X. Zhao Y. (2023). Rg3-loaded P407/CS/HA hydrogel inhibits UVB-induced oxidative stress, inflammation and apoptosis in HaCaT cells. Biomed. Pharmacother. Sep. 165, 115177. 10.1016/j.biopha.2023.115177 37467650

[B8] Ding R. Kan Q. Wang T. Xiao R. Song Y. Li D. (2024). Ginsenoside Rh2 regulates triple-negative breast cancer proliferation and apoptosis via the IL-6/JAK2/STAT3 pathway. Front. Pharmacol. 15, 1483896. 10.3389/fphar.2024.1483896 39845783 PMC11751231

[B9] Fan H. Wang Y. Zhang X. Chen J. Zhou Q. Yu Z. (2019). Ginsenoside compound K ameliorates imiquimod-induced psoriasis-like dermatitis through inhibiting REG3A/RegIIIγ expression in keratinocytes. Biochem. Biophys. Res. Commun. 515, 665–671. 10.1016/j.bbrc.2019.06.007 31182284

[B10] Fan W. Fan L. Wang Z. Mei Y. Liu L. Li L. (2024). Rare ginsenosides: a unique perspective of ginseng research. J. Adv. Res. 66, 303–328. 10.1016/j.jare.2024.01.003 38195040 PMC11674801

[B11] Feng L. Liu X. Sun K. Sun Y. Wu W. Chen C. (2024). Ginsenoside Rb1 inhibits the proliferation of lung cancer cells by inducing the mitochondrial-mediated apoptosis pathway. Anticancer Agents Med. Chem. 24, 928–941. 10.2174/0118715206299212240304142047 38465430

[B12] Ferrara F. Verduci C. Laconi E. Mangione A. Dondi C. Del Vecchio M. (2024). Current therapeutic overview and future perspectives regarding the treatment of psoriasis. Int. Immunopharmacol. 143, 113388. 10.1016/j.intimp.2024.113388 39405929

[B13] Gao Y. Li J. Wang J. Li X. Li J. Chu S. (2020). Ginsenoside Rg1 prevent and treat inflammatory diseases: a review. Int. Immunopharmacol. Oct. 87, 106805. 10.1016/j.intimp.2020.106805 32731179

[B14] Geng X. Wang J. Liu Y. Liu L. Liu X. Zhao Y. (2024). Research progress on chemical diversity of saponins in Panax ginseng. Chin. Herb. Med. 16, 529–547. 10.1016/j.chmed.2024.08.005 39606259 PMC11589341

[B15] Go G. Y. Jo A. Seo D. W. Kim W. Y. Kim Y. K. So E. Y. (2020). Ginsenoside Rb1 and Rb2 upregulate Akt/mTOR signaling-mediated muscular hypertrophy and myoblast differentiation. J. Ginseng Res. May 44, 435–441. 10.1016/j.jgr.2019.01.007 32372865 PMC7195574

[B16] Grabowska K. Galanty A. Pecio Ł. Stojakowska A. Malarz J. Żmudzki P. (2024). Selectivity screening and structure–cytotoxic activity observations of selected oleanolic acid (OA)-type saponins from the amaranthaceae family on a wide panel of human cancer cell lines. Molecules 29, 3794. 10.3390/molecules29163794 39202875 PMC11357256

[B17] Guo C. Huang Q. Wang Y. Yao Y. Li J. Chen J. (2023). Therapeutic application of natural products: NAD+ metabolism as potential target. Phytomedicine 114, 154768. 10.1016/j.phymed.2023.154768 36948143

[B18] Guo J. Zhang H. Lin W. Lu L. Su J. Chen X. (2023). Signaling pathways and targeted therapies for psoriasis. Signal Transduction Targeted Therapy 8, 437. 10.1038/s41392-023-01655-6 38008779 PMC10679229

[B19] Han M. J. Kim D. H. (2020). Effects of red and fermented ginseng and ginsenosides on allergic disorders. Biomolecules 10, 10. 10.3390/biom10040634 32326081 PMC7226199

[B20] Han Q. Han L. Tie F. Wang Z. Ma C. Li J. (2020). (20S)‐Protopanaxadiol ginsenosides induced cytotoxicity *via* blockade of autophagic flux in HGC‐27 cells. Chem. and Biodivers. 17, e2000187. 10.1002/cbdv.202000187 32384197

[B21] Han N. R. Ko S. G. Moon P. D. Park H. J. (2021). Ginsenoside Rg3 attenuates skin disorders *via* down-regulation of MDM2/HIF1α signaling pathway. J. Ginseng Res. Sep. 45, 610–616. 10.1016/j.jgr.2021.06.008 34803431 PMC8587510

[B22] He B. Chen D. Zhang X. Yang R. Yang Y. Chen P. (2022). Oxidative stress and ginsenosides: an update on the molecular mechanisms. Oxidative Medicine Cellular Longevity 2022, 9299574. 10.1155/2022/9299574 35498130 PMC9045968

[B23] Hill D. A. Spergel J. M. (2018). The atopic march: critical evidence and clinical relevance. Ann. Allergy Asthma Immunol. Feb 120, 131–137. 10.1016/j.anai.2017.10.037 29413336 PMC5806141

[B24] Hou J. Kim S. (2018). Possible role of ginsenoside Rb1 in skin wound healing *via* regulating senescent skin dermal fibroblast. Biochem. Biophys. Res. Commun. 499, 381–388. 10.1016/j.bbrc.2018.03.170 29577907

[B25] Hou M. Wang R. Zhao S. Wang Z. (2021). Ginsenosides in panax genus and their biosynthesis. Acta Pharm. Sin. B 11, 1813–1834. 10.1016/j.apsb.2020.12.017 34386322 PMC8343117

[B26] Huang W. C. Huang T. H. Yeh K. W. Chen Y. L. Shen S. C. Liou C. J. (2021). Ginsenoside Rg3 ameliorates allergic airway inflammation and oxidative stress in mice. J. Ginseng Res. Nov. 45, 654–664. 10.1016/j.jgr.2021.03.002 34764720 PMC8569325

[B27] Huang M. Y. Chen Y. C. Lyu W. Y. He X. Y. Ye Z. H. Huang C. Y. (2023). Ginsenoside Rh2 augmented anti-PD-L1 immunotherapy by reinvigorating CD8(+) T cells *via* increasing intratumoral CXCL10. Pharmacol. Res. Dec 198, 106988. 10.1016/j.phrs.2023.106988 37984507

[B28] Huo D. S. Zhang M. Cai Z. P. Dong C. X. Wang H. Yang Z. J. (2015). The role of nerve growth factor in ginsenoside Rg1-Induced regeneration of injured rat sciatic nerve. J. Toxicol. Environ. Health A 78, 1328–1337. 10.1080/15287394.2015.1085943 26529404

[B29] Huynh D. T. N. Baek N. Sim S. Myung C. S. Heo K. S. (2020). Minor ginsenoside Rg2 and Rh1 attenuates LPS-induced acute liver and kidney damages *via* downregulating activation of TLR4-STAT1 and inflammatory cytokine production in macrophages. Int. J. Mol. Sci. Sep. 11, 21. 10.3390/ijms21186656 32932915 PMC7555743

[B30] Hwang S. J. Bang H. J. Lee H. J. (2023). Ginsenoside re inhibits melanogenesis and melanoma growth by downregulating microphthalmia-associated transcription factor. Biomed. Pharmacother. Sep. 165, 115037. 10.1016/j.biopha.2023.115037 37393867

[B31] Jang I. S. Jo E. Park S. J. Baek S. J. Hwang I. H. Kang H. M. (2020). Proteomic analyses reveal that ginsenoside Rg3(S) partially reverses cellular senescence in human dermal fibroblasts by inducing peroxiredoxin. J. Ginseng Res. Jan. 44, 50–57. 10.1016/j.jgr.2018.07.008 32148389 PMC7033328

[B32] Jia W. Zhang H. Cheng J. Zhang X. Han C. He Y. (2025). Extraction, preparation, pharmacological activities, and potential applications of ginsenosides Rk1 and Rg5. J. Ethnopharmacol. 355, 120678. 10.1016/j.jep.2025.120678 41052692

[B33] Jiang M. Chi J. Qiao Y. Wang J. Zhang Z. Liu J. (2025). Ginsenosides Rg1, Rb1 and rare ginsenosides: promising candidate agents for parkinson's disease and Alzheimer's disease and network pharmacology analysis. Pharmacol. Res. 212, 107578. 10.1016/j.phrs.2025.107578 39756554

[B34] Kim M.-Y. Cho J. Y. (2013). 20S-dihydroprotopanaxadiol, a ginsenoside derivative, boosts innate immune responses of monocytes and macrophages. J. Ginseng Res. 37, 293–299. 10.5142/jgr.2013.37.293 24198654 PMC3818955

[B35] Kim J. S. Joo E. J. Chun J. Ha Y. W. Lee J. H. Han Y. (2012). Induction of apoptosis by ginsenoside Rk1 in SK-MEL-2-human melanoma. Arch. Pharm. Res. Mar. 35, 717–722. 10.1007/s12272-012-0416-0 22553065

[B36] Kim J. H. Yi Y. S. Kim M. Y. Cho J. Y. (2017). Role of ginsenosides, the main active components of Panax ginseng, in inflammatory responses and diseases. J. Ginseng Res. Oct. 41, 435–443. 10.1016/j.jgr.2016.08.004 29021688 PMC5628327

[B37] Kim J. H. Bae Y. C. Lee J. W. Choi J. S. Bae S. H. (2019). Effects of ginsenoside Rg3 on apoptosis in A375.S2 melanoma cells. Transl. Cancer Res. Apr 8, 357–366. 10.21037/tcr.2018.11.15 35116768 PMC8797360

[B38] Kim J.-H. Lee R. Hwang S.-H. Choi S.-H. Kim J.-H. Cho I.-H. (2024). Ginseng and ginseng byproducts for skincare and skin health. J. Ginseng Res. 48, 525–534. 10.1016/j.jgr.2024.09.006 39583168 PMC11583465

[B39] Ko E. Park S. Lee J. H. Cui C. H. Hou J. Kim M. H. (2019). Ginsenoside Rh2 ameliorates atopic dermatitis in NC/Nga mice by suppressing NF-kappaB-Mediated thymic stromal lymphopoietin expression and T helper type 2 differentiation. Int. J. Mol. Sci. Dec 4, 20. 10.3390/ijms20246111 31817146 PMC6940811

[B40] Kong L. Liu Y. Li J. Wang Y. Ji P. Shi Q. (2024). Ginsenoside Rg1 alleviates chronic inflammation-induced neuronal ferroptosis and cognitive impairments *via* regulation of AIM2-Nrf2 signaling pathway. J. Ethnopharmacology 330, 118205. 10.1016/j.jep.2024.118205 38641079

[B41] Lee H.-J. Kim M. (2023). Challenges and future trends in the treatment of psoriasis. Int. Journal Molecular Sciences 24, 13313. 10.3390/ijms241713313 37686119 PMC10487560

[B42] Li Q. Zhai C. Wang G. Zhou J. Li W. Xie L. (2021). Ginsenoside Rh1 attenuates ovalbumin-induced asthma by regulating Th1/Th2 cytokines balance. Biosci. Biotechnol. Biochem. 85, 1809–1817. 10.1093/bbb/zbab099 34057179

[B43] Li D. Luo Z. B. Zhu J. Wang J. X. Jin Z. Y. Qi S. (2024). Ginsenoside F2-Mediated intestinal microbiota and its metabolite propionic acid positively impact the gut-skin axis in atopic dermatitis mice. J. Agric. Food Chem. Jan. 10 (72), 339–350. 10.1021/acs.jafc.3c06015 38150707

[B44] Li J. K. Jiang X. L. Zhang Z. Chen W. Q. Peng J. J. Liu B. (2024). 20(S)-Ginsenoside Rh2 induces apoptosis and autophagy in melanoma cells *via* suppressing Src/STAT3 signaling. J. Ginseng Res. Nov. 48, 559–569. 10.1016/j.jgr.2024.07.002 39583170 PMC11583474

[B45] Li Y. Yang K. Zhao L. Xu C. Zhou W. Wang Z. (2024). Effects of schisandra lignans on the absorption of protopanaxadiol-type ginsenosides mediated by P-glycoprotein and protopanaxatriol-type ginsenosides mediated by CYP3A4. J. Ethnopharmacol. 318, 117057. 10.1016/j.jep.2023.117057 37597677

[B46] Li Z.-Y. Dai Y.-x. Wu Z.-m. Li G. Pu P.-m. Hu C.-w. (2024). Network pharmacology analysis and animal experiment validation of neuroinflammation inhibition by total ginsenoside in treating CSM. Phytomedicine 126, 155073. 10.1016/j.phymed.2023.155073 38417244

[B47] Li Y. Zhang G. Wang J. Li J. Liu Y. Pan S. (2025). The biological functions of ginsenoside and its applications in animal husbandry. Front. Veterinary Sci. 12, 1648629. 10.3389/fvets.2025.1648629 40979376 PMC12446036

[B48] Liu Y. Fan D. (2020). The preparation of ginsenoside Rg5, its antitumor activity against breast cancer cells and its targeting of PI3K. Nutr. Jan. 18, 12. 10.3390/nu12010246 31963684 PMC7019936

[B49] Liu X. Y. Xiao Y. K. Hwang E. Haeng J. J. Yi T. H. (2019). Antiphotoaging and antimelanogenesis properties of ginsenoside C-Y, a ginsenoside Rb2 metabolite from American ginseng PDD-ginsenoside. Photochem Photobiol. Nov. 95, 1412–1423. 10.1111/php.13116 31074886

[B50] Liu L. Xu F.-R. Wang Y.-Z. (2020). Traditional uses, chemical diversity and biological activities of panax L.(Araliaceae): a review. J. Ethnopharmacology 263, 112792. 10.1016/j.jep.2020.112792 32311488

[B51] Liu X. Jiang Y. Fu W. Yu X. Sui D. (2020). Combination of the ginsenosides Rb3 and Rb2 exerts protective effects against myocardial ischemia reperfusion injury in rats. Int. J. Mol. Med. 45, 519–531. 10.3892/ijmm.2019.4414 31789417 PMC6984776

[B52] Liu E. Gao H. Zhao Y. Pang Y. Yao Y. Yang Z. (2022). The potential application of natural products in cutaneous wound healing: a review of preclinical evidence. Front. Pharmacol. 13, 900439. 10.3389/fphar.2022.900439 35935866 PMC9354992

[B53] Liu X. Y. Li H. Hwang E. Park B. Xiao Y. K. Liu S. (2022). Chemical distance measurement and system pharmacology approach uncover the novel protective effects of biotransformed ginsenoside C-Mc against UVB-irradiated photoaging. Oxid. Med. Cell Longev. 2022, 4691576. 10.1155/2022/4691576 35186187 PMC8850047

[B54] Liu. Y. Qu L. Wan S. Li Y. Fan D. (2022). Ginsenoside Rk1 prevents UVB irradiation-mediated oxidative stress, inflammatory response, and collagen degradation *via* the PI3K/AKT/NF-κB pathway *in vitro* and *in vivo* . J. Agric. Food Chem. 70, 15804–15817. 10.1021/acs.jafc.2c06377 36472249

[B55] Liu R. Zhang B. Zou S. Cui L. Lin L. Li L. (2024). Ginsenoside Rg1 induces autophagy in colorectal cancer through inhibition of the Akt/mTOR/p70S6K pathway. J. Microbiol. Biotechnol. 28 (34), 774–782. 10.4014/jmb.2310.10043 38668684 PMC11091659

[B56] Liu S. Wang M. Xiao H. Ye J. Cao L. Li W. (2024). Advancements in research on the effects of panax notoginseng saponin constituents in ameliorating learning and memory disorders. Heliyon 15 (10), e28581. 10.1016/j.heliyon.2024.e28581 38586351 PMC10998096

[B57] Lorz L. R. Kim M. Y. Cho J. Y. (2020). Medicinal potential of Panax ginseng and its ginsenosides in atopic dermatitis treatment. J. Ginseng Res. 44, 8–13. 10.1016/j.jgr.2018.12.012 32095092 PMC7033350

[B58] Luo C. Xu X. Wei X. Feng W. Huang H. Liu H. (2019). Natural medicines for the treatment of fatigue: bioactive components, pharmacology, and mechanisms. Pharmacol. Res. 148, 104409. 10.1016/j.phrs.2019.104409 31446039

[B59] Lv D. L. Chen L. Ding W. Zhang W. Wang H. L. Wang S. (2018). Ginsenoside G-Rh2 synergizes with SMI-4a in anti-melanoma activity through autophagic cell death. Chin. Med. 13, 11. 10.1186/s13020-018-0168-y 29483938 PMC5820787

[B60] Mao J. Ma X. Zhu J. Zhang H. (2022). Ginsenoside Rg1 ameliorates psoriasis-like skin lesions by suppressing proliferation and NLRP3 inflammasomes in keratinocytes. J. Food Biochem. 46, e14053. 10.1111/jfbc.14053 35218026

[B61] Marshall R. Moor J. Birch-Machin M. (2025). BC02 clinical demonstration and biomarker study of skin ageing, monitoring and intervention. Br. J. Dermatology 193, ljaf085.215. 10.1093/bjd/ljaf085.215

[B62] Meng L. Ji R. Dong X. Xu X. Xin Y. Jiang X. (2019). Antitumor activity of ginsenoside Rg3 in melanoma through downregulation of the ERK and Akt pathways. Int. J. Oncol. 54, 2069–2079. 10.3892/ijo.2019.4787 31081060 PMC6521931

[B63] Minh Nguyen H. Truong Nguyen H. Win N. Piow Wong C. Vu Huynh K. L. Hoang N. N. (2020). Antimelanogenic activity of ocotillol-type saponins from Panax vietnamensis. Chem. Biodivers. 17, e2000037. 10.1002/cbdv.202000037 32163220

[B64] Mohanan P. Subramaniyam S. Mathiyalagan R. Yang D. C. (2018). Molecular signaling of ginsenosides Rb1, Rg1, and Rg3 and their mode of actions. J. Ginseng Res. Apr 42, 123–132. 10.1016/j.jgr.2017.01.008 29719458 PMC5926405

[B65] Moratilla-Rivera I. Sánchez M. Valdés-González J. A. Gómez-Serranillos M. P. (2023). Natural products as modulators of Nrf2 signaling pathway in neuroprotection. Int. J. Mol. Sci. Feb 13, 24. 10.3390/ijms24043748 36835155 PMC9967135

[B66] Nguyen H. M. Nguyen H. T. Win N. Wong C. P. Huynh K. L. V. Hoang N. N. (2020). Antimelanogenic activity of ocotillol-type saponins from Panax vietnamensis. 10.1002/cbdv.202000037 32163220

[B67] Oh J. M. Yoon H. Joo J. Y. Im W. T. Chun S. (2024). Therapeutic potential of ginseng leaf extract in inhibiting mast cell-mediated allergic inflammation and atopic dermatitis-like skin inflammation in DNCB-treated mice. Front. Pharmacol. 15, 1403285. 10.3389/fphar.2024.1403285 38841363 PMC11150533

[B68] Osada-Oka M. Hirai S. Izumi Y. Misumi K. Samukawa K. Tomita S. (2018). Red ginseng extracts attenuate skin inflammation in atopic dermatitis through p70 ribosomal protein S6 kinase activation. J. Pharmacol. Sci. 136, 9–15. 10.1016/j.jphs.2017.11.002 29274665

[B69] Park S. Y. Park J. H. Kim H. S. Lee C. Y. Lee H. J. Kang K. S. (2018). Systems-level mechanisms of action of panax ginseng: a network pharmacological approach. J. Ginseng Res. Jan. 42, 98–106. 10.1016/j.jgr.2017.09.001 29348728 PMC5766701

[B70] Park N. J. Bong S. K. Lee S. Jung Y. Jegal H. Kim J. (2020). Compound K improves skin barrier function by increasing SPINK5 expression. J. Ginseng Res. Nov. 44, 799–807. 10.1016/j.jgr.2019.11.006 33192123 PMC7655487

[B71] Peng Y. Zhang R. Yang X. Zhang Z. Kang N. Bao L. (2019). Ginsenoside Rg3 suppresses the proliferation of prostate cancer cell line PC3 through ROS-induced cell cycle arrest. Oncol. Lett. 17, 1139–1145. 10.3892/ol.2018.9691 30655875 PMC6312957

[B72] Piao M. J. Kang K. A. Zhen A. X. Fernando P. Ahn M. J. Koh Y. S. (2019). Particulate matter 2.5 mediates cutaneous cellular injury by inducing mitochondria-associated endoplasmic reticulum stress: protective effects of ginsenoside Rb1. Antioxidants (Basel). 9, 8. 10.3390/antiox8090383 31505827 PMC6769862

[B73] Qi Z. Chen L. Li Z. Shao Z. Qi Y. Gao K. (2019). Immunomodulatory effects of (24R)-Pseudo-Ginsenoside HQ and (24S)-Pseudo-Ginsenoside HQ on cyclophosphamide-induced immunosuppression and their anti-tumor effects study. Int. J. Mol. Sci. 15, 20. 10.3390/ijms20040836 30769948 PMC6413033

[B74] Qiao J. Zhao Y. Liu Y. Zhang S. Zhao W. Liu S. (2022). Neuroprotective effect of ginsenoside re against neurotoxin-induced parkinson's disease models *via* induction of Nrf2. Mol. Med. Rep. 25, 215. 10.3892/mmr.2022.12731 35543148 PMC9133950

[B75] Qin Y. Zhang Z. Jiang R. (2025). Ginsenoside Rg1 promotes wound healing in mice with superficial second-degree burns through energy metabolism, cell migration, and cell adhesion pathways. J. Med. Food 28, 165–173. 10.1089/jmf.2024.k.0146 39469786

[B76] Qu Z. Y. Zong Y. Zheng P. H. Jin Y. P. Li W. Wang H. C. (2021). New malonylginsenosides from the fresh fruits of Panax notoginseng. Fitoterapia. 150, 104844. 10.1016/j.fitote.2021.104844 33548359

[B77] Rendon A. Schäkel K. (2019). Psoriasis pathogenesis and treatment. Int. J. Mol. Sci. 23, 20. 10.3390/ijms20061475 30909615 PMC6471628

[B78] Samukawa K. Izumi Y. Shiota M. Nakao T. Osada-Oka M. Miura K. (2012). Red ginseng inhibits scratching behavior associated with atopic dermatitis in experimental animal models. J. Pharmacol. Sci. 118, 391–400. 10.1254/jphs.11182fp 22382656

[B79] Shan X. Aziz F. Tian L. L. Wang X. Q. Yan Q. Liu J. W. (2015). Ginsenoside Rg3-induced EGFR/MAPK pathway deactivation inhibits melanoma cell proliferation by decreasing FUT4/LeY expression. Int. J. Oncol. 46, 1667–1676. 10.3892/ijo.2015.2886 25672851 PMC6903901

[B80] Shi Q. He Q. Chen W. Long J. Zhang B. (2019). Ginsenoside Rg1 abolish imiquimod‐induced psoriasis‐like dermatitis in BALB/c mice *via* downregulating NF‐κB signaling pathway. J. Food Biochemistry 43, e13032. 10.1111/jfbc.13032 31502279

[B81] Shibata S. (2001). Chemistry and cancer preventing activities of ginseng saponins and some related triterpenoid compounds. J. Korean Medical Science 16, S28–S37. 10.3346/jkms.2001.16.S.S28 11748374 PMC3202208

[B82] Shin K. O. Choe S. J. Uchida Y. Kim I. Jeong Y. Park K. (2018). Ginsenoside Rb1 enhances keratinocyte migration by a Sphingosine-1-Phosphate-Dependent mechanism. J. Med. Food 21, 1129–1136. 10.1089/jmf.2018.4246 30148701 PMC6913107

[B83] Shin M.-S. Song J. H. Choi P. Lee J. H. Kim S.-Y. Shin K.-S. (2018). Stimulation of innate immune function by Panax ginseng after heat processing. J. Agric. Food Chem. 66, 4652–4659. 10.1021/acs.jafc.8b00152 29659255

[B84] Song X. Wang L. Fan D. (2022). Insights into recent studies on biotransformation and pharmacological activities of ginsenoside Rd. Biomolecules 28, 12. 10.3390/biom12040512 35454101 PMC9031344

[B85] Song Y. Chen C. Li W. (2024). Ginsenoside Rb(1) in cardiovascular and cerebrovascular diseases: a review of therapeutic potentials and molecular mechanisms. Chin. Herb. Med. Oct. 16, 489–504. 10.1016/j.chmed.2024.09.006 39606264 PMC11589305

[B86] Spencer R. K. Jin J. Q. Elhage K. G. Davis M. S. Hakimi M. Bhutani T. (2023). Comparative efficacy of biologics and oral agents in palmoplantar psoriasis and palmoplantar pustulosis: a systematic review and network meta-analysis of randomized clinical trials. J. Am. Acad. Dermatology 89, 423–425. 10.1016/j.jaad.2023.04.043 37121476

[B87] Sroka-Tomaszewska J. Trzeciak M. (2021). Molecular mechanisms of atopic dermatitis pathogenesis. Int. J. Mol. Sci. 16, 22. 10.3390/ijms22084130 33923629 PMC8074061

[B88] Tang M. Bian W. Cheng L. Zhang L. Jin R. Wang W. (2018). Ginsenoside Rg3 inhibits keloid fibroblast proliferation, angiogenesis and collagen synthesis *in vitro via* the TGF-β/Smad and ERK signaling pathways. Int. J. Mol. Med. 41, 1487–1499. 10.3892/ijmm.2018.3362 29328420 PMC5819908

[B89] Tang J. R. Chen G. Lu Y. C. Tang Q. Y. Song W. L. Lin Y. (2021). Identification of two UDP-glycosyltransferases involved in the main oleanane-type ginsenosides in Panax japonicus var. major. Planta 253, 91. 10.1007/s00425-021-03617-0 33818668

[B90] Tian T. Ko C. N. Luo W. Li D. Yang C. (2023). The anti-aging mechanism of ginsenosides with medicine and food homology. Food Funct. 14, 9123–9136. 10.1039/d3fo02580b 37766674

[B91] Tian Z. F. Hu R. Y. Wang Z. Wang Y. J. Li W. (2025). Molecular mechanisms behind the inhibitory effects of ginsenoside Rg3 on hepatic fibrosis: a review. Arch. Toxicol. 99, 541–561. 10.1007/s00204-024-03941-w 39729114

[B92] Tímár J. Ladányi A. (2022). Molecular pathology of skin melanoma: epidemiology, differential diagnostics, prognosis and therapy prediction. Int. J. Mol. Sci. 11, 23. 10.3390/ijms23105384 35628196 PMC9140388

[B93] Wan S. Liu Y. Shi J. Fan D. Li B. (2021). Anti-photoaging and anti-inflammatory effects of ginsenoside Rk3 during exposure to UV irradiation. Front. Pharmacol. 12, 716248. 10.3389/fphar.2021.716248 34671254 PMC8521102

[B94] Wan X. Jin X. Wu X. Dong D. Yang H. Tan R. (2024). Ginsenoside Rd reduces cell proliferation of non-small cell lung cancer cells by p53-mitochondrial apoptotic pathway. Heliyon 15 (10), e32483. 10.1016/j.heliyon.2024.e32483 38933967 PMC11201117

[B95] Wang Y. Liu Y. Zhang X.-Y. Xu L.-H. Ouyang D.-Y. Liu K.-P. (2014). Ginsenoside Rg1 regulates innate immune responses in macrophages through differentially modulating the NF-κB and PI3K/Akt/mTOR pathways. Int. Immunopharmacology 23, 77–84. 10.1016/j.intimp.2014.07.028 25179784

[B96] Wang M. Yan S. J. Zhang H. T. Li N. Liu T. Zhang Y. L. (2017). Ginsenoside Rh2 enhances the antitumor immunological response of a melanoma mice model. Oncol. Lett. 13, 681–685. 10.3892/ol.2016.5490 28356946 PMC5351349

[B97] Wang C. Liu J. Deng J. Wang J. Weng W. Chu H. (2020). Advances in the chemistry, pharmacological diversity, and metabolism of 20 (R)-ginseng saponins. J. Ginseng Research 44, 14–23. 10.1016/j.jgr.2019.01.005 32095093 PMC7033361

[B98] Wang J. Zeng L. Zhang Y. Qi W. Wang Z. Tian L. (2022). Pharmacological properties, molecular mechanisms and therapeutic potential of ginsenoside Rg3 as an antioxidant and anti-inflammatory agent. Front. Pharmacol. 13, 975784. 10.3389/fphar.2022.975784 36133804 PMC9483152

[B99] Wang Y. Wu J. Hong Y. Zhu J. Zhang Y. Zhang J. (2024). Ginsenosides retard atherogenesis *via* remodelling host–microbiome metabolic homeostasis. Br. J. Pharmacol. 181, 1768–1792. 10.1111/bph.16320 38355288

[B100] Wang W. Xu X. Yang M. Jiang M. Wang D. Tang C. (2024). Ginsenoside compound K reduces psoriasis-related inflammation by activation of the glucocorticoid receptor in keratinocytes. Curr. Mol. Pharmacol. 17, e18761429254358. 10.2174/0118761429254358231120135400 38389423

[B101] Wang L. Wu X. Wang X. Dong M. Zhang H. Zhao P. (2025). Targeting CHEK1: ginsenosides-Rh2 and Cu2O@ G-Rh2 nanoparticles in thyroid cancer. Cell Biol. Toxicol. 41, 30. 10.1007/s10565-024-09961-7 39808342 PMC11732901

[B102] Wang Y. Bu P. Deng Y. Zhao W. Pan G. (2025). Research progress on traditional Chinese medicine compounds in autoimmune-related skin diseases. Front. Immunol. 16, 1629288. 10.3389/fimmu.2025.1629288 41112251 PMC12529447

[B103] Wang J. Xu D. Lai Y. Zhao Y. Jin Q. Yin Y. (2026). Ginsenoside re ameliorates UVB-induced skin photodamage by modulating the glutathione metabolism pathway: insights from integrated transcriptomic and metabolomic analyses. Int. J. Mol. Sci. 27, 708. 10.3390/ijms27020708 41596360 PMC12841036

[B104] Wu Y. Wang X. Q. Wu J. Y. Chen Y. J. Bai J. X. Li A. S. (2025). A tri-compound formula comprising ginsenoside Rg1, tetrandrine and icariin alleviates atopic dermatitis symptoms in a mouse model. Phytomedicine. 141, 156737. 10.1016/j.phymed.2025.156737 40222169

[B105] Xin C. Kim J. Quan H. Yin M. Jeong S. Choi J.-I. (2019). Ginsenoside Rg3 promotes Fc gamma receptor-mediated phagocytosis of bacteria by macrophages *via* an extracellular signal-regulated kinase 1/2 and p38 mitogen-activated protein kinase-dependent mechanism. Int. Immunopharmacology 77, 105945. 10.1016/j.intimp.2019.105945 31644962

[B106] Yang J. Li X. Sun T. Gao Y. Chen Y. Jin Y. (2016). Semisynthesis and bioactive evaluation of oxidized products from 20 (S)-ginsenoside Rg3, Rh2, protopanaxadiol (PPD) and their 20 (R)-epimers as cytotoxic agents. Steroids 106, 26–34. 10.1016/j.steroids.2015.12.005 26703442

[B107] Yang W. S. Yi Y.-S. Kim D. Kim M. H. Park J. G. Kim E. (2017). Nuclear factor kappa-B-and activator protein-1-mediated immunostimulatory activity of compound K in monocytes and macrophages. J. Ginseng Res. 41, 298–306. 10.1016/j.jgr.2016.06.004 28701870 PMC5489765

[B108] Yang J. Zhang L. Peng X. Zhang S. Sun S. Ding Q. (2023). Polymer-based wound dressings loaded with ginsenoside Rg3. Molecules 28, 5066. 10.3390/molecules28135066 37446725 PMC10343326

[B109] Yang C. Qian C. Zheng W. Dong G. Zhang S. Wang F. (2024). Ginsenoside Rh2 enhances immune surveillance of natural killer (NK) cells *via* inhibition of ERp5 in breast cancer. Phytomedicine 123, 155180. 10.1016/j.phymed.2023.155180 38043385

[B110] Yang L. Wang W. Zhang A. (2025). PARP1 promoter hypermethylation promotes arsenic-induced skin damage by driving telomere dysfunction-mediated keratinocyte senescence. Ecotoxicol. Environ. Saf. 304, 119108. 10.1016/j.ecoenv.2025.119108 41027193

[B111] Yi Y.-S. (2021). New mechanisms of ginseng saponin-mediated anti-inflammatory action *via* targeting canonical inflammasome signaling pathways. J. Ethnopharmacol. 278, 114292. 10.1016/j.jep.2021.114292 34089812

[B112] Yin S. Xia F. Zou W. Jiang F. Shen K. Sun B. (2024). Ginsenoside Rg1 regulates astrocytes to promote angiogenesis in spinal cord injury *via* the JAK2/STAT3 signaling pathway. J. Ethnopharmacol. 334, 118531. 10.1016/j.jep.2024.118531 38971343

[B113] You L. Cha S. Kim M.-Y. Cho J. Y. (2022). Ginsenosides are active ingredients in Panax ginseng with immunomodulatory properties from cellular to organismal levels. J. Ginseng Research 46, 711–721. 10.1016/j.jgr.2021.12.007 36312737 PMC9597430

[B114] Yu K. Chen F. Li C. (2012). Absorption, disposition, and pharmacokinetics of saponins from Chinese medicinal herbs: what do we know and what do we need to know more? Curr. Drug Metabolism 13, 577–598. 10.2174/1389200211209050577 22292787

[B115] Yu T. Tang Y. Zhang F. Zhang L. (2023). Roles of ginsenosides in sepsis. J. Ginseng Res. 47, 1–8. 10.1016/j.jgr.2022.05.004 36644389 PMC9834008

[B116] Zhan Z. Zhang J. Huang W. Huang J. (2025). Transcriptomic strategy provides molecular insights into the growth and ginsenosides accumulation of Panax ginseng. Phytomedicine 143, 156834. 10.1016/j.phymed.2025.156834 40440906

[B117] Zhang H. Su Y. Sun Z. Chen M. Han Y. Li Y. (2021). Ginsenoside Rg1 alleviates Aβ deposition by inhibiting NADPH oxidase 2 activation in APP/PS1 mice. J. Ginseng Res. Nov. 45, 665–675. 10.1016/j.jgr.2021.03.003 34764721 PMC8569324

[B118] Zhang C. Merana G. R. Harris-Tryon T. Scharschmidt T. C. (2022). Skin immunity: dissecting the complex biology of our body's outer barrier. Mucosal Immunology 15, 551–561. 10.1038/s41385-022-00505-y 35361906

[B119] Zhang J. Zhao R. Hou G. Wang Q. Zhao F. Liu Z. (2023). Stereoscopic differences in the identification, bioactivity, and metabolism of C-20 and C-24 epimeric ginseng saponins. Mini Rev. Med. Chem. 23, 804–820. 10.2174/1389557522666221012095258 36237162

[B120] Zhang J. Li Z. Song X. Cai P. Liu Q. (2025). Ginsenoside CK and retinol on UVA-induced photoaging exert the synergistic effect through antioxidant and antiapoptotic mechanisms. Sci. Rep. 15, 16664. 10.1038/s41598-025-99304-1 40360842 PMC12075579

[B121] Zheng S. W. Xiao S. Y. Wang J. Hou W. Wang Y. P. (2019). Inhibitory effects of ginsenoside Ro on the growth of B16F10 melanoma *via* its metabolites. Mol. Aug 17, 24. 10.3390/molecules24162985 31426477 PMC6721120

[B122] Zheng Y. Tan H. Chai J. Han L. Zhai C. Lee J. (2024). Ginseng fruit rare saponins (GFRS) improved inflammatory response: *in vitro* and *in vivo* assessment. Fitoter. Dec 179, 106244. 10.1016/j.fitote.2024.106244 39396651

[B123] Zhou J. Gao Y. Yi X. Ding Y. (2015). Ginsenoside Rh2 suppresses neovascularization in xenograft psoriasis model. Cell Physiol. Biochem. 36, 980–987. 10.1159/000430272 26087848

